# Indoor residual spraying with a mixture of clothianidin (a neonicotinoid insecticide) and deltamethrin provides improved control and long residual activity against pyrethroid resistant *Anopheles gambiae* sl in Southern Benin

**DOI:** 10.1371/journal.pone.0189575

**Published:** 2017-12-18

**Authors:** Corine Ngufor, Augustin Fongnikin, Mark Rowland, Raphael N’Guessan

**Affiliations:** 1 London School of Hygiene and Tropical Medicine (LSHTM), London, United Kingdom; 2 Centre de Recherches Entomologiques de Cotonou (CREC), Cotonou, Benin; Universidade Federal do Rio de Janeiro, BRAZIL

## Abstract

**Introduction:**

There is an urgent need for new insecticides for indoor residual spraying (IRS) which can provide improved and prolonged control of malaria vectors that have developed resistance to existing insecticides. The neonicotinoid, clothianidin represents a class of chemistry new to public health. Clothianidin acts as an agonist on nicotinic acetyl choline receptors. IRS with a mixture of Clothianidin and another WHO approved insecticide such as deltamethrin could provide improved control of insecticide resistant malaria vector populations and serve as a tool for insecticide resistance management.

**Methods:**

The efficacy and residual activity of a novel IRS mixture of deltamethrin and clothianidin was evaluated against wild pyrethroid resistant *An*. *gambiae* sl in experimental huts in Cove, Benin. Two application rates of the mixture were tested and comparison was made with clothianidin and deltamethrin applied alone. To assess the residual efficacy of the treatments on different local wall substrates, the inner walls of the experimental huts were covered with either cement, mud or plywood.

**Results:**

Clothianidin demonstrated a clear delayed expression in mortality of wild pyrethroid resistant *An*. *gambiae* sl in the experimental huts which reached its full effect 120 hours after exposure. Overall mortality over the 12-month hut trial was 15% in the control hut and 24–29% in the deltamethrin-treated huts. The mixture of clothianidin 200mg/m^2^ and deltamethrin 25mg/m^2^ induced high overall hut mortality rates (87% on mud walls, 82% on cement walls and 61% on wooden walls) largely due to the clothianidin component and high hut exiting rates (67–76%) mostly due to the deltamethrin component. Mortality rates remained >80% for 8–9 months on mud and cement walls. The residual activity trend was confirmed by results from monthly in situ cone bioassays with laboratory susceptible *An*. *gambiae* Kisumu strain.

**Conclusion:**

IRS campaigns with the mixture of clothianidin plus deltamethrin have the potential to provide prolonged control of malaria transmitted by pyrethroid resistant mosquito populations.

## Background

With increased funding and impetus from the Global Fund and the US President’s Malaria Initiative (PMI), the use of indoor residual spraying (IRS) for malaria vector control in Africa has increased significantly in recent years [1]. This together with a massive scale up in the distribution of LLINs has contributed significantly to the substantial reductions in malaria morbidity and mortality seen today [1]. Twelve insecticides belonging to four classes of chemistry can be used for IRS [2]. Apart from DDT which lasts over 6 months most WHO-recommended IRS insecticides have a short residual life (2–6 months) on local wall substrates and thus would require multiple application rounds in holoendemic areas where transmission occurs year-round. This poses a major challenge to the effective use of IRS in most of sub-Saharan Africa given the operational resources required for the implementation of re-current IRS campaigns.

Of the four classes of insecticides approved for IRS, pyrethoids have been the most widely used owing to their low cost, low mammalian toxicity and efficacy [3]. However, resistance to pyrethroids and DDT is now widespread across sub-Saharan Africa [3, 4] and this has led to an increased interest in the use of carbamate and organophosphate insecticides within the existing portfolio of WHO approved insecticides for IRS [5, 6]. Nevertheless, resistance to organophosphates and carbamates especially due to the insensitive acetyl cholinesterase gene (Ace1R) has been reported in several areas in West Africa and is spreading [7–10]. The identification of novel long-lasting IRS insecticides which do not show any cross resistance to current insecticides is thus vital.

Clothiandin is a neonicotinoid insecticide which represents a new class of chemistry for public health. Neonicotinoids are widely used in crop protection against piercing/sucking insects and in veterinary medicine against cat and dog fleas [11]. They exhibit great biological efficacy against a broad spectrum of insect pests among the Hemiptera, Coleoptera, Thysanoptera, Lepidoptera and Diptera. Clothianidin acts as an agonist on nicotinic actetylcholine receptors thus demonstrating a different mode of action to pyrethroids, organophosphates and carbamates. Because neonicotinoids have very low affinity for vertebrate nicotinic receptors relative to insect nicotinic receptors, they generally show very low toxicity to mammals [12, 13] and are thus promising for public health use. Considering the novel mode of action, they show potential to provide improved control of vector populations that have developed resistance to existing public health insecticides.

The use of mixtures of unrelated insecticides in co-formulations for IRS has the potential to improve malaria vector control through the differential effects of the insecticides involved and manage insecticide resistance in areas where resistance to either active ingredient is not yet established [4]. The latter is based on the concept that insect vectors which are resistant to one component of the mixture can be killed by the other; thus, preventing selection of insecticide resistant genotypes [10, 14]. Because IRS targets the insect vector at a stage when it is more likely to make longer contact with the insecticide (fed and resting on the wall), the use of mixtures for IRS could be very effective.

In this study, we evaluated the efficacy and residual activity of a mixture of deltamethrin and clothiandin against wild pyrethroid resistant *An gambiae* in experimental huts in Cove, Benin. Two application rates of the mixture were tested and comparison was made with the two insecticides applied alone. To assess the residual efficacy of the treatments on different local wall substrates, the inner walls of the experimental huts were covered with either cement, mud or plywood.

## Material and methods

### Study site and experimental huts

The study was performed in a newly constructed experimental hut station in Cove, Central Benin belonging to the LSHTM/CREC Collaborative Research Programme which is being used for the evaluation of several indoor vector control tools to WHOPES Phase 2 standards [15–17]. The station is situated at the center of a large rice growing field. The rice paddies provide extensive breeding sites for *An gambiae* sl throughout the year. The vector population consists of both *An coluzzi* and *An gambiae ss* with the latter occurring at much lower frequencies (~23%) and mostly in the drier seasons. The rainy season extends from March to October and the dry season from November to February. The local vector population is highly resistant to pyrethroids. Molecular genotyping and microarray studies showed that pyrethroid resistance in the Cove population is mediated by high levels of L1014F gene (90%) and overexpression of metabolic genes including CYP6P3, a P450 which metabolizes pyrethroids [16].

The trial was carried out over 12 months between December 2013 and November 2014 in 11 experimental huts of the WHO-approved West African design [18]. The experimental huts are built on concrete plinths surrounded by water-filled moats to prevent entry of scavenging ants, and have veranda traps to capture the exiting mosquitoes. They are made of brick plastered with cement on the inside, with a corrugated iron roof. The huts have a ceiling of palm thatch and four window slits (1cm gap) on the walls through which mosquitoes enter.

### Susceptibility tests

To determine the frequency of resistance to deltamethrin in the wild *An gambiae* sl Cove strain during the trial, mosquitoes which emerged from larvae collected from breeding sites close to the experimental hut station were tested in WHO resistance kits lined with deltamethrin 0.05% treated papers. Approximately 100 unfed 2–5 day old female mosquitoes were exposed for 1 hour in batches of 25 mosquitoes. Comparison was made with the laboratory susceptible *An gambiae* Kisumu strain. Mortality was scored 24 h later.

### Experimental hut treatments

The following insecticides were compared in the experimental huts:

- Deltamethrin (K-Othrine) WG 25
- Clothianidin WG 70
- Clothianidin plus Deltamethrin mixture prepared by mixing both insecticides in the spray tank

To assess the residual efficacy of the above on different local wall substrates, the insecticides/mixture were applied as IRS in huts with either cement, dried mud or plywood interior walls. The following 11 treatments and application rates were thus applied in 11 experimental huts:

- Unsprayed control hut—cement walled,
- Deltamethrin 25 mg/m^2^ (DM25)—one cement, one mud and one plywood walled hut,
- Clothianidin 200 mg/m^2^ (CT200)—cement walled hut,
- Clothianidin 100 mg/m^2^ + Deltamethrin 25 mg/m^2^ mixture (CT100+DM25)—one cement, one mud and one plywood walled hut,
- Clothianidin 200 mg/m^2^ + Deltamethrin 25 mg/m^2^ mixture (CT200+DM25)—one cement, one mud and one plywood walled hut.

The walls and ceiling of each experimental hut was sprayed using a Hudson XPert compression sprayer. The mixtures were prepared by mixing the appropriate amount of each insecticide in the spray tank. To improve spraying accuracy, spray swaths were marked out on the interior hut walls prior to spraying and a guidance pole was attached to the end of the spray lance to maintain a fixed distance to the wall.

### Hut trial procedure

The trial followed WHOPES guidelines [19]. Treatments were randomly allocated to the experimental huts. Eleven consenting adult human volunteers slept in the huts from 20:00 to 05:00 each night of the study to attract mosquitoes into the hut and were rotated between huts on successive nights to adjust for any variation in individual attractiveness to mosquitoes. In the morning of each day of the trial, mosquitoes were collected from the rooms and verandahs of the hut and brought to the laboratory where they were identified and scored for blood feeding status and mortality. Mortality was recorded for up to 120 hours across all the hut treatments including the control.

The efficacy of each treatment in the experimental huts was thus expressed in terms of:

- Exiting rates: proportion of mosquitoes collected in the verandah of a treated hut relative to the proportion in the control hut.
- Blood-feeding rates: proportion of mosquitoes that were blood fed in each hut.
- Mortality: proportion of mosquitoes found dead after 120 h holding time.

#### Residual activity of insecticide treatments

To assess residual activity of the insecticide treatments on the different substrates, 2–5 days old mosquitoes of the susceptible *An*. *gambiae* Kisumu laboratory strain were tested in WHO cone bioassays in situ on the interior treated walls of each hut 3 days after application of the treatment and subsequently every month of the trial. A total of 50 mosquitoes were tested per hut in batches of 10 mosquitoes per cone and one cone was tested per wall/ceiling surface. Mosquitoes were exposed for 30 min according to WHO IRS guidelines [19]. Knockdown was recorded after 60 minutes and mortality after a 120-hour holding period.

### Data analysis

#### Statistical analysis

Proportional outcomes (blood-feeding, exiting and mortality) related to each experimental hut treatment were assessed using binomial generalized linear mixed models (GLMMs) with a logit link function, fitted using the ‘lme4’ package for R (version 2.15.0). A separate model was fitted for each outcome. In addition to the fixed effect of each treatment, each model included random effects to account for the following sources of variation: between the 11 huts; between the 11 sleepers; between the months of the trial; and finally, an observation-level random effect to account for variation not explained by the other terms in the model (over dispersion).

Differences in deterrence, personal protection and mass killing effect between the treatments was analysed using negative binomial regression based on numbers entering, blood-feeding and killed respectively with adjustment for the abovementioned covariates.

### Ethical considerations

The study was approved by the Ethics Review Committee of the London School of Hygiene & Tropical Medicine and the Ministry of Health in Benin. Human volunteer sleepers who slept in the huts to attract mosquitoes gave informed consent prior to their participation and were provided chemoprophylaxis. Through the course of the study, the sleepers were examined regularly for signs of fever by a stand-by nurse; any sleepers testing positive for malaria were withdrawn from the study and treated properly. Permission to use the experimental hut station was obtained from Centre de Recherche Entomologique de Cotonou.

## Results

### Susceptibility tests

Knock down and mortality rates of the susceptible laboratory *An gambiae* Kisumu strain after exposure to deltamethrin 0.05% treated papers were 98% and 100% respectively. With the wild *An gambiae* sl from Cove, knockdown and mortality were 11% and 8% respectively (Table 1) showing that the wild strain had a high frequency of resistance to deltamethrin.

**Table 1:** Efficacy of deltamethrin-treated papers (0.05%) against adult *An gambiae* from Cove (wild) and Kisumu (laboratory susceptible) in WHO cylinder bioassays.

Strains	N exposed	% Knockdown (95% CI)	% Mortality (95% CI)
*An gambiae* Kisumu	102	98 (95–100)	100 (95–100)
*An gambiae* sl Cove (wild)*	104	11 (5–17)	8 (3–15)

*samples were collected as larvae from breeding sites close to the experimental huts in Cove during the trial

### Experimental hut results

The overall experimental hut results obtained over 12 months of the trial are presented in Table 2. The primary experimental hut data are provided in S1 Table (1–6 months) and S2 Table (7–12 months).

**Table 2:** Results with wild pyrethroid resistant *An gambiae* sl entering IRS treated experimental huts in Cove, Benin.

	Total collected	% Exiting (95% CI)	% Blood fed (95% CI)	% Corrected 120h mortality (95% CI)
**Cement Huts**				
Untreated	11154	43 (42–44)^a^	87 (86–88)^a^	-
DM25	9505	67 (66–68)^b^	83 (82–84)^b^	13 (12–14)^a^
CT100 + DM25	7999	68 (67–69)^b^	84 (83–85)^b^	62 (61–63)^b^
CT200 + DM25	8166	67 (65–68)^b^	82 (81–83)^b^	79 (78–80)^c^
CT200	6972	39 (37–40)^c^	84 (83–85)^b^	89 (88–90)^d^
**Mud Huts**				
DT 25	6231	69 (68–70)^b^	86 (85–87)^ac^	19 (18–20)^e^
CT100 + DM25	7429	74 (73–75)^d^	84 (83–85)^b^	72 (71–73)^f^
CT200 + DM25	7393	72 (71–73)^d^	82 (81–83)^b^	85 (84–86)^g^
**Wood Huts**				
DM25	7288	77 (76–78)^e^	88 (87–89)^a^	19 (18–20)^e^
CT100 + DM25	7108	76 (75–76)^e^	85 (84–86)^bc^	46 (45–47)^h^
CT200 + DM25	7387	73 (72–74)^d^	86 (85–87)^ac^	62 (61–63)^i^

Values bearing the same letter superscript along a column are not significantly different at the 5% level (P>0.05), DM = deltamethrin, CT = clothianidin.

#### Overall hut entry and exiting

The average total number of mosquitoes collected per hut treatment over the course of the 12-month trial was 7,876 (Table 2). A significantly higher number of mosquitoes entered the control hut compared to the treated huts (Table 2; P<0.005). The proportion exiting the control hut was 43%. With all hut wall substrates, exiting rates with deltamethrin (67–77%) and the clothianidin + deltamethrin mixtures (67–76%), were consistently and significantly higher than the control hut (43%; P<0.05) and the hut treated with clothianidin alone (39%, P<0.05). Exiting rates did not differ considerably between the deltamethrin treated huts and the mixture huts for all three types of hut wall substrates. These results showed that treatment induced exiting from the mixture-treated huts was largely due to the deltamethrin component.

#### Overall blood feeding rates

Blood feeding rates were generally very high across all treatments (ranging between 82 and 88%) and did not show much difference relative to the control (87%). No significant difference in blood feeding rates was also detected between huts with the higher and lower dose mixtures for any of the wall substrates (P>0.05). With cement walled huts, blood feeding rates did not differ significantly between deltamethrin and the mixtures (P>0.05). However, the deltamethrin showed a very small increase in blood feeding rates in mud and plywood walled huts relative to the mixtures (P<0.05).

#### Mortality rates

**Fig 1:**
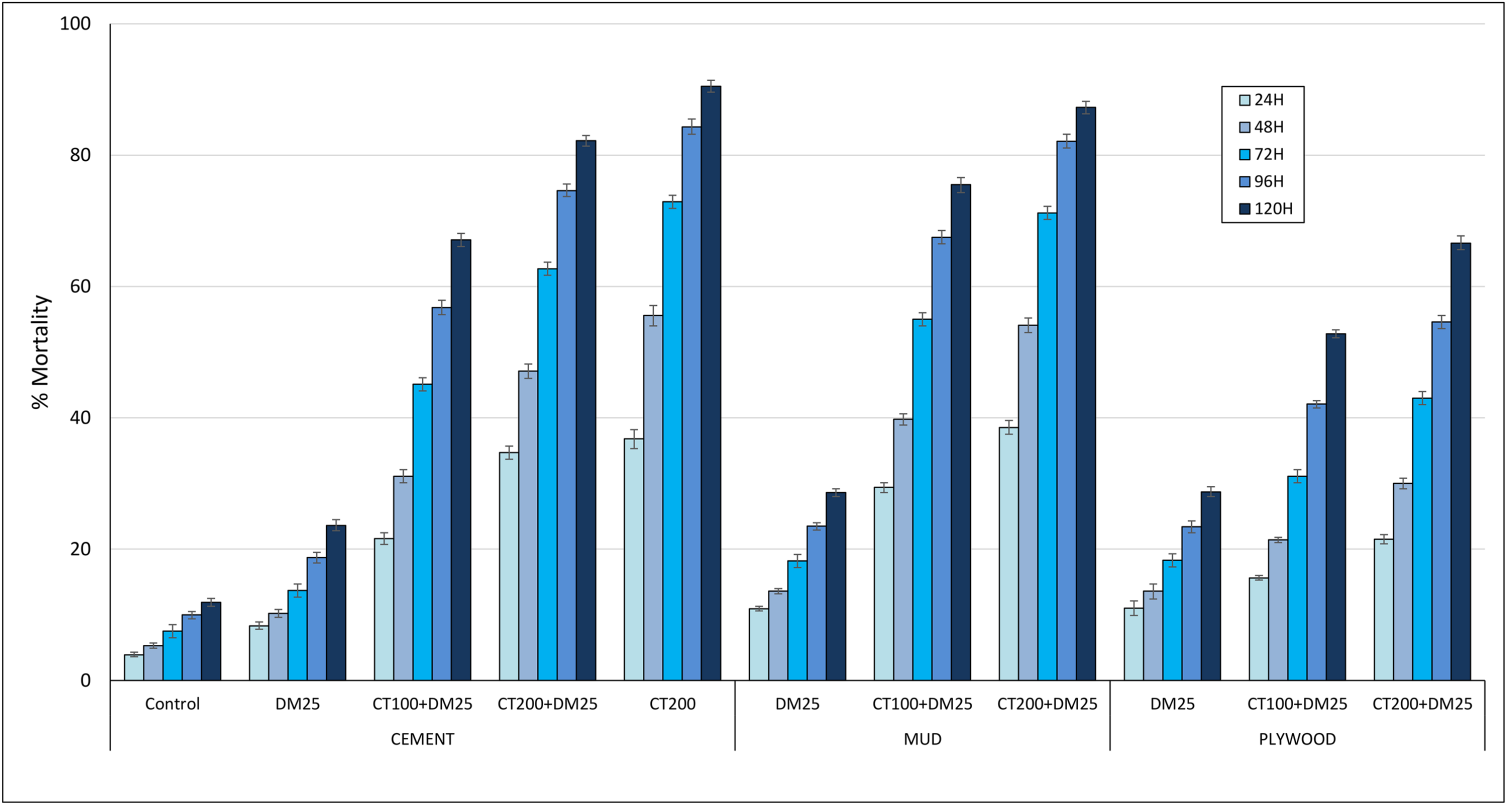
Delayed mortality (%) of wild pyrethroid resistant *An gambiae* in IRS treated experimental huts in Cove, Benin with cement, mud and plywood walls. Each bar represents % overall mortality after 24, 48, 72, 96 and 120 hours for each hut wall substrate and each treatment. Error bars represent 95% confidence intervals. DM = deltamethrin, CT = clothianidin.

Delayed mortality: Fig 1 presents the overall mortality rates recorded after 24, 48, 72, 96 and 120 hours in huts with cement, mud and plywood walls. Mortality with the control was <5% after 24h and increased to only 12% after 120h. For all hut wall substrates, clothianidin whether applied alone or as a mixture with deltamethrin demonstrated a delayed expression in mortality with a full effect showing only after 5 days of observation. For example, mortality in cement walls treated with clothianidin alone increased steadily from 38% after 24h to 91% after 120 hours (Fig 1). This trend of delayed mortality was not strongly expressed in huts treated with deltamethrin alone; mortality did not exceed 29% after 120h.

**Fig 2:**
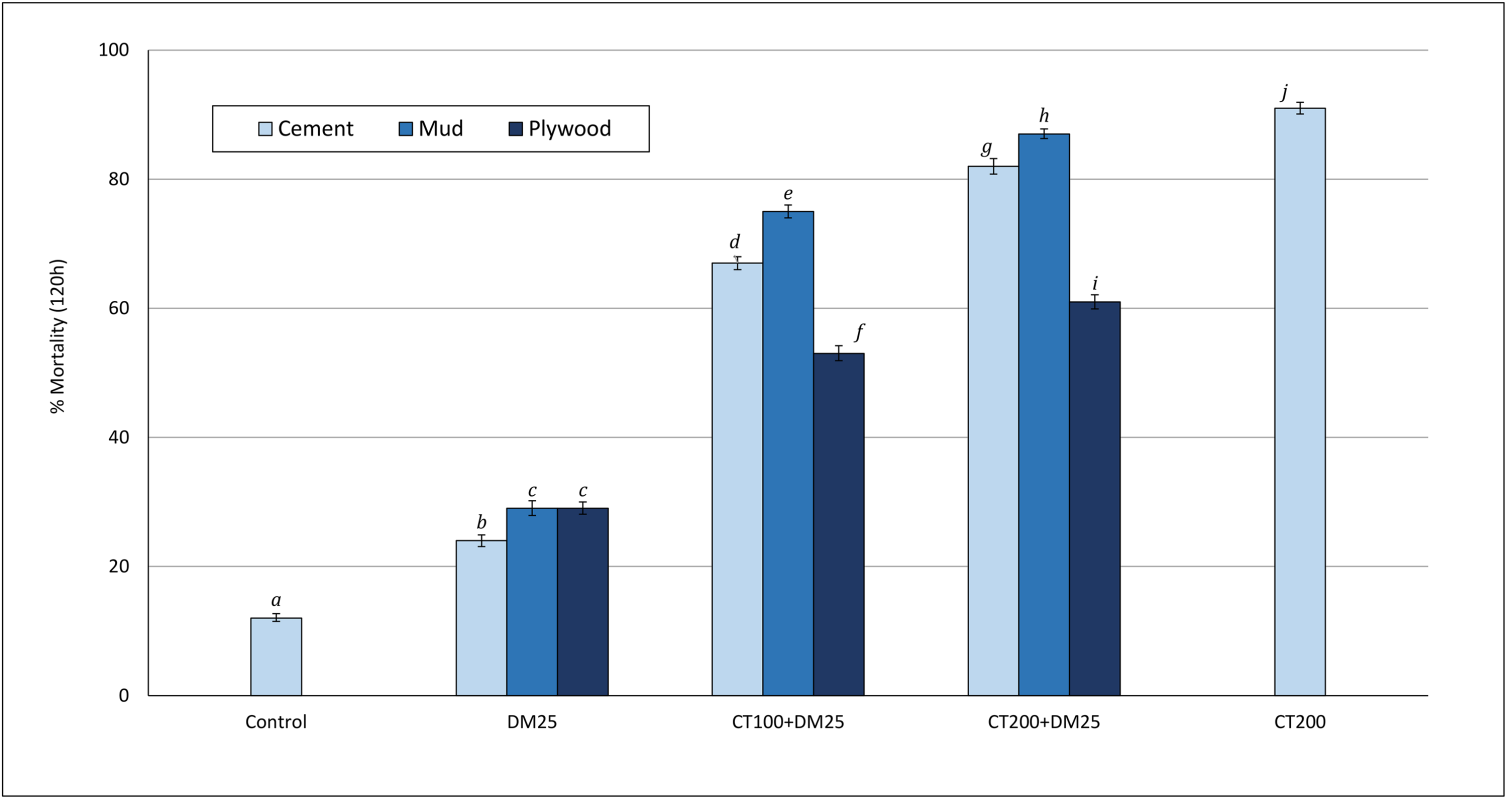
Overall mortality rates of wild pyrethroid resistant *An gambiae* in cement, mud and plywood walled experimental huts in Cove, Benin. Each bar represents overall % mortality (120h) for each wall substrate and each treatment over 12 months. Bars bearing the same letter label are not significantly different at the 5% level; P>0.05. Error bars represent 95% confidence intervals. DM = deltamethrin, CT = clothianidin.

Overall mortality after 12 months: The overall mortality rates (recorded after 120 hours) over the 12-month trial are presented in Fig 2. Overall mortality was 12% in the control hut and < 30% in deltamethrin treated huts (24% with cement walled hut and 29% each with mud and plywood walled huts). Mortality rates were generally higher with the mixtures relative to deltamethrin alone (51%–82% vs. 24–29%; P<0.05). For each hut wall substrate, proportions dead were also generally higher in the hut with the higher dose mixture (CT200+DM25) relative to the hut with the lower dose mixture (CT100+DM25). In the cement-walled huts for example, mortality was 67% with the CT100+DM25 mixture and 82% with the cloth200+delta25 mixture (P<0.001). The mixtures killed higher proportions of mosquitoes in mud walled huts (75% with CT100+DM25 and 87% with CT200+DM25) relative to cement walled huts (67% with CT100+DM25 and 82% with CT200+DM25; P<0.05) and plywood walled huts (53% with CM100+DM25 and 61% with CT200+DM25; P<0.05). The highest overall mortality rate was achieved in the cement-walled hut treated with clothianidin alone (91%).

#### Residual efficacy over 12 months

**Fig 3:**
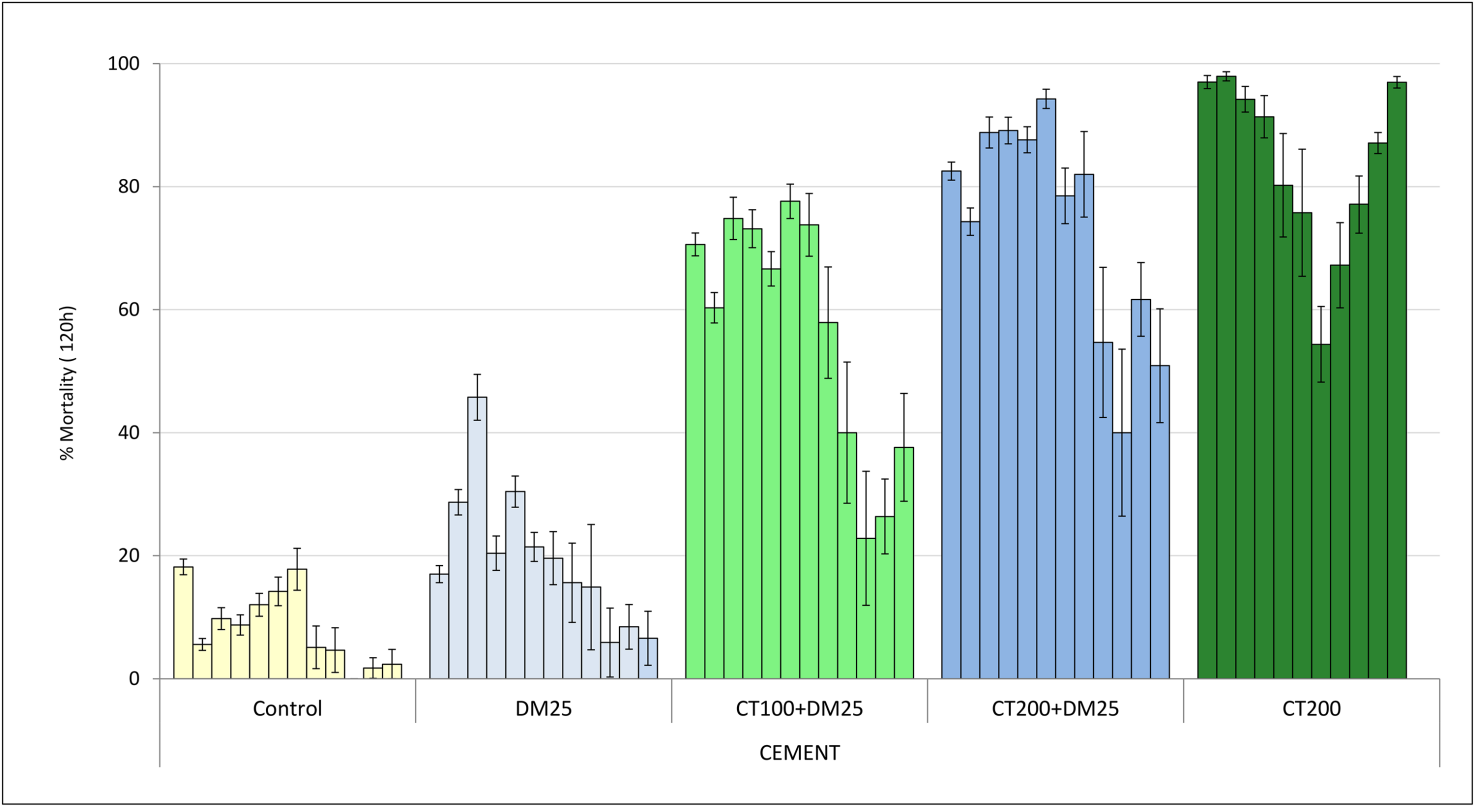
Monthly mortality rates of wild free-flying pyrethroid resistant *An*. *gambiae* entering IRS treated cement-walled experimental huts in Cove, Benin. Each bar represents % mortality (120h) over each successive month of the trial for treatments applied in CEMENT plastered huts. Error bars represent 95% confidence intervals. DM = deltamethrin, CT = clothianidin.

**Fig 4:**
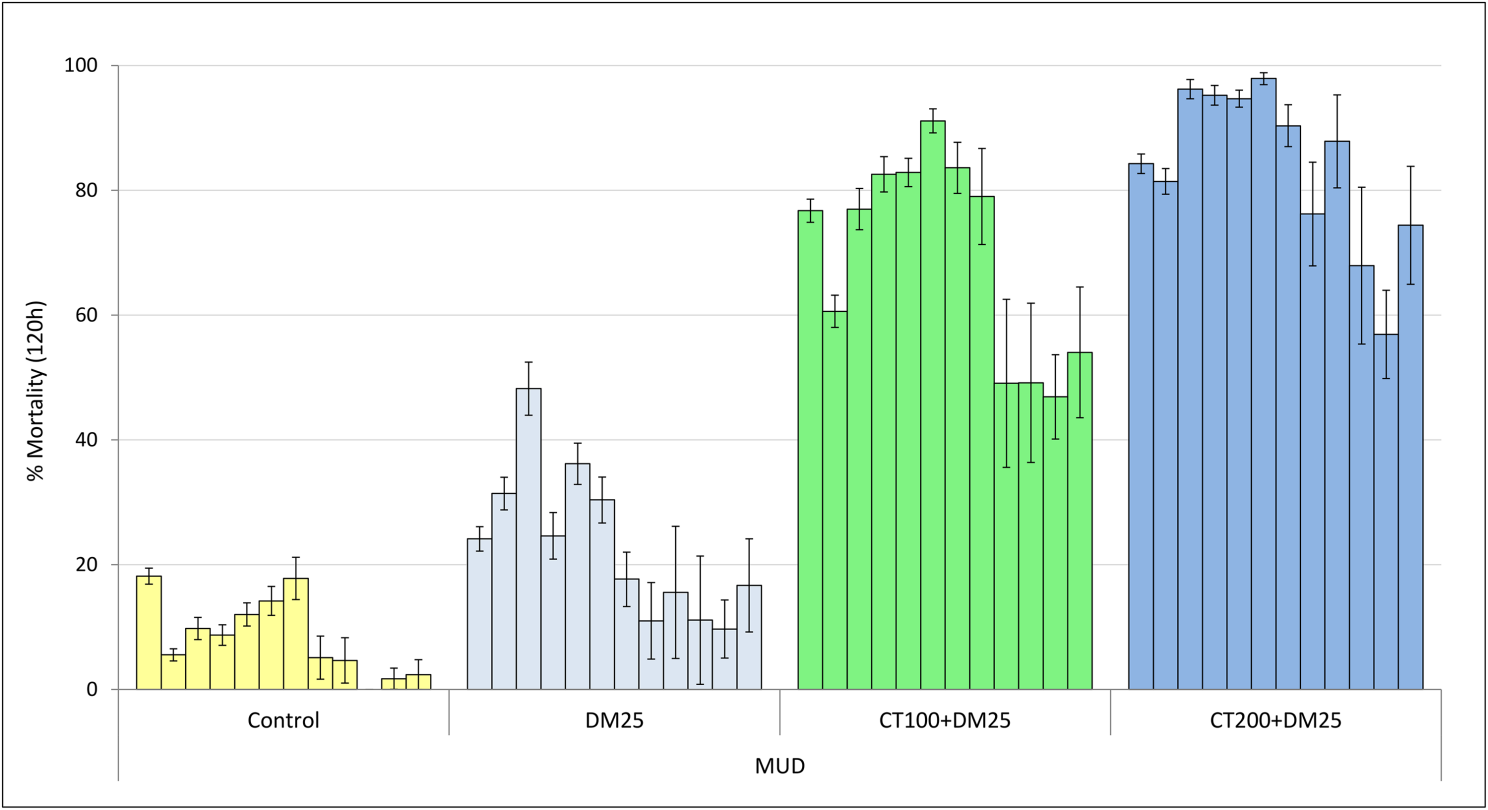
Monthly mortality rates of wild free-flying pyrethroid resistant *An*. *gambiae* entering IRS treated cement-walled experimental huts in Cove, Benin. Each bar represents % mortality (120h) over each successive month of the trial for treatments applied in MUD plastered huts. Error bars represent 95% confidence intervals. DM = deltamethrin, CT = clothianidin.

**Fig 5:**
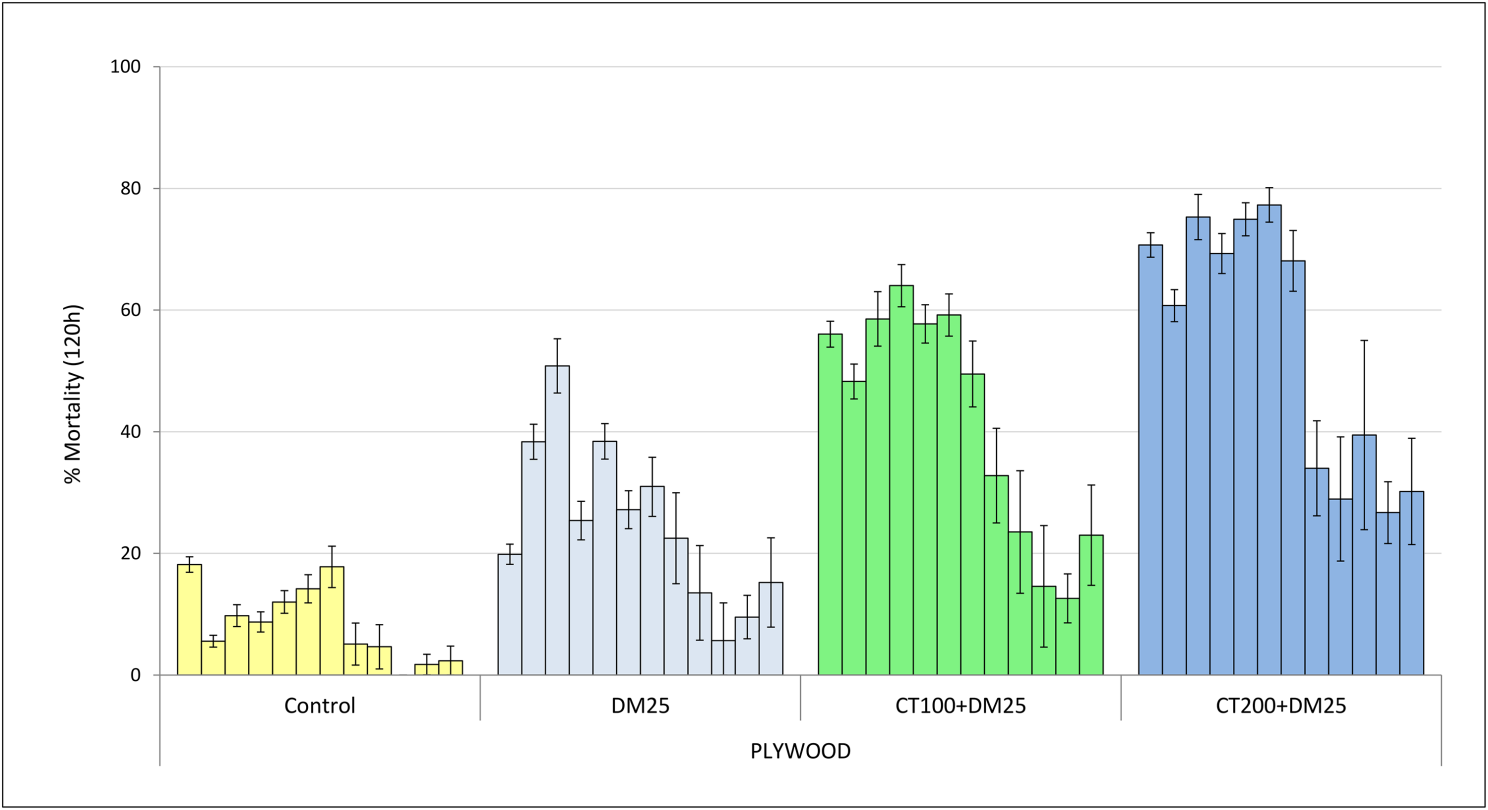
Monthly mortality rates of wild free-flying pyrethroid resistant *An*. *gambiae* entering IRS treated cement-walled experimental huts in Cove, Benin. Each bar represents % mortality (120h) over each successive month of the trial for treatments applied in WOODEN-walled huts. Error bars represent 95% confidence intervals. DM = deltamethrin, CT = clothianidin.

The monthly mortality rates of wild free flying *An gambiae* sl which entered the experimental huts during the course of the 12-month trial are presented in Figs 3, 4 and 5 for cement, mud and wooden wall huts respectively.

Cement walled huts: In cement walled huts the lower dose mixture (CT100 + DM25) killed 60–80% of wild pyrethroid resistant *An gambiae* sl entering the huts for the first 7 months after which mortality declined progressively. A longer-lasting trend in mortality was observed in the cement walled hut treated with the higher dose mixture (CT200 + DM25) compared to the low dose mixture (P<0.05); mortality was > 80% for the first 8 months of the trial and declined to 40–60% in the last 4 months. Mortality in the cement walled hut treated with clothianidin alone was the more persistent but also more variable; mortality was >80% for the first 5 months and then varied between 54% and 97% for the rest of the trial. The proportion killed in the deltamethrin treated cement hut was 28–45% in the first 3 months and declined rapidly thereafter.

Mud walled huts: Mortality in mud walled huts with both low and high dose mixtures was >80% for the first 7 to 8 months respectively. After this period, mortality declined more steeply in the lower dose mixture (45–55%) but less so in the higher dose mixture (55–75%). The data showed that the rate of decay of the mixtures was slower in the mud walled huts relative to the cement and plywood walled huts (P<0.001).

Wooden walled huts: In the wooden walled huts the higher dose mixture (CT200+DM25) killed more mosquitoes in the first 7 months of the trial (60–78%) compared to the lower dose mixture (CT100+DM25) (48–65%) (P<0.005). After this period, mortality rates declined steeply in both mixture treatments. The trend in monthly mortality in the deltamethrin treated plywood walled hut was similar to that in the cement and mud walled huts.

#### Residual bioassay activity

**Fig 6:**
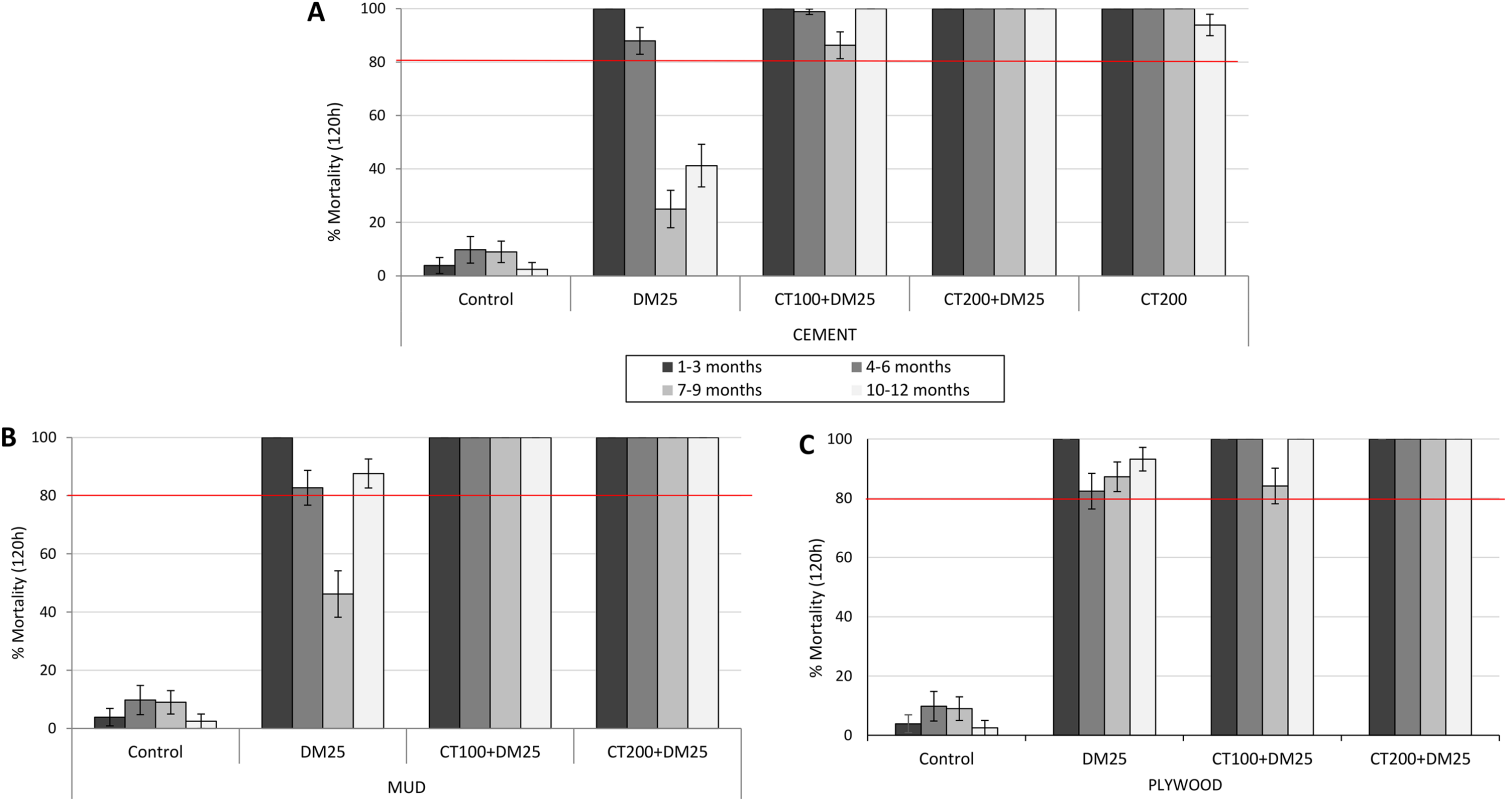
Residual efficacy of IRS treatments on (A) cement walled, (B) mud-walled and (C) plywood-walled experimental huts in Cove, Benin: Cone bioassay mortality with laboratory susceptible *An*. *gambiae* Kisumu. Each bar represents average mortality over 3 successive monthly in situ cone bioassays. Error bars represent 95% confidence intervals. DM = deltamethrin, CT = clothianidin.

The quarterly residual mortality rates (120h post-exposure) of susceptible *An gambiae* Kisumu laboratory strain exposed in monthly *in situ* cone bioassays to the different hut treatments are presented in Fig 6A, 6B and 6C for cement, mud and plywood walls respectively. Cone bioassay mortality on the control hut walls did not exceed 10% in any quarter of the trial. With the mixtures and clothianidin treatments, cone bioassay mortality was very high (>90%) through all 4 quarters of the trial irrespective of the wall substrate (Fig 6). Cone bioassay mortality with deltamethrin on cement walls was >80% for the first 2 quarters but declined very steeply to <40% thereafter (Fig 6A). A similar trend was observed with deltamethrin treated mud walls, however, mortality increased to 75% in the last 3 months of the trial (Fig 6B). Deltamethrin induced more persistent mortality on plywood walls; cone bioassay mortality was >80% in all quarters of the trial (Fig 6C).

## Discussion

The objective of the current study was to assess the efficacy of an insecticide mixture of clothianidin and deltamethrin for IRS against free-flying pyrethroid resistant malaria vectors. The results showed significantly improved and prolonged mortality rates in huts treated with the mixture and clothianidin alone compared to deltamethrin alone and this can be largely attributed to the novel mode of action of clothianidin. The neonicotinoid therefore represents a very promising insecticide for IRS campaigns in areas where malaria vectors have developed resistance to current insecticides.

The mortality rates recorded with clothianidin whether alone or in the mixture clearly demonstrated a delayed effect lasting up to 5 days. Neonicotinoids by acting on the insect nicotinic actetylcholine receptor exhibit both acute contact toxicity and stomach poison [20] and the latter could be responsible for the delayed effect on mortality of mosquitoes. Previous studies on fruit flies also demonstrated a significant increase in mortality with clothianidin when observation time increased from 24h to 72h [21]. This delayed effect has also been observed in a similar parallel study evaluating the efficacy of clothianidin and deltamethrin IRS mixture against multiple insecticide resistant malaria vectors in Cote D’Ivoire (unpublished data) thus confirming our findings. The results therefore demonstrate that failure to assess delayed mortality could lead to an underestimation of the efficacy of neonicotinoids in evaluation studies. This could explain the low toxicity reported with the neonicotinoid dinotefuran, in a previous study testing its efficacy against various mosquito strains [22] where adult mortality was recorded only for 24hours.

Since pyrethroids have been the most widely used insecticides for malaria vector control for decades, WHOPES guidelines were tailored towards the evaluation of fast-acting insecticides (like pyrethroids), hence the guidelines state that mortality rates should be recorded within 24 hours post-exposure [19]. The identification of new active ingredients for malaria vector control poses a major challenge to chemical companies, it is therefore important that the guidelines used for their evaluation are adapted to the mode of action of the insecticide being tested.

The high blood-feeding rates are generally expected with IRS treatments as there is no physical barrier to prevent mosquitoes from feeding; mosquitoes would usually feed on the sleeper before resting on the IRS-treated wall to digest their blood meal. Apart from mortality, treatment-induced early exophily and deterrence of mosquitoes from treated home structures are other important insecticidal characteristics desired of IRS applications which reduce vector-human contact thus reducing vectorial capacity. While exiting rates were low in the huts with clothianidin alone, very high exiting rates were recorded in huts with the mixtures and this could be attributed to the excito-repellent effect of the pyrethroid component in the mixture. Hence the mixture induced improved levels of mortality due to the clothianidin component and high exophily from the huts due to the pyrethroid component thus providing some justification for its use over clothianidin alone or deltamethrin alone. Due to a potential hut position effect and the inability to rotate IRS treatments, it was not possible to effectively assess the deterrent effect of the IRS treatments.

Because the current study was designed as a proof of concept to demonstrate the efficacy of an IRS mixture of clothianidin and deltamethrin, the mixture was prepared as a tank mix of both formulations prior to the hut applications. The WHO however recommends against the use of *ad hoc* mixtures for IRS; for maximum impact, mixtures should ideally be co-formulated into a single product to facilitate application by field operators and ensure that both active ingredients decay at the same rate [4]. Based on the positive results from the current preliminary study, Bayer has formulated the mixture into a single wettable powder product in water soluble sachets containing 500g/kg of clothianidin and 62.5g/kg of deltamethrin also known as Fludora^®^. Studies are underway to assess the efficacy of Fludora^®^ against pyrethroid resistant malaria vectors in WHOPES Phase II experimental hut studies and Phase III community randomized controlled trials in several countries across East and West Africa.

Although neonicotinoids have not been used in public health, they are widely applied in agricultural operations in sub-Saharan Africa. There is increasing evidence of the link between agricultural use of insecticides and selection of resistance to these insecticides in mosquitoes in the region [23]. Modelling studies have suggested that slow/late life acting insecticides like clothianidin could impose less selection pressure for resistance compared to faster acting insecticides [24]. Based on simulation studies [14], it is also expected that the clothianidin and deltamethrin mixture if deployed in areas where resistance to either active ingredient is not yet established, will slow down the development of resistance to clothianidin in malaria vectors and better delay the spread of pyrethroid resistance compared to clothianidin alone or deltamethrin alone. Further studies in areas with low to moderate levels of pyrethroid resistance should be performed to assess the resistance management potential of this new mixture for IRS. Modelling studies have indicated that for mixtures to be effective it is important that both components are applied at the recommended operational dose [14, 25]. The results showed greater persistence in mortality with the higher dose mixture relative to the lower dose mixture thus suggesting that the clothianidin 200 + deltamethrin 25 mg/m^2^ mixture would be a more appropriate dose to achieve effective and prolonged control of pyrethroid resistant mosquitoes and for resistance management.

While the mixture induced considerably high (60–80%) and long residual mortality against wild pyrethroid resistant mosquitoes on all wall substrates, this effect lasted longest on mud walls (9 months) compared to cement and wooden walls (6–8 months). Most IRS insecticides tested on a variety of wall substrates have shown significantly longer residual activity on cement walls than on mud walls [26–28]. The results therefore demonstrate that the clothianidin and deltamethrin WP mixture may be a more suitable insecticide for IRS campaigns in most rural endemic areas in sub-Saharan Africa where most houses have mud walls. The reasons behind this longer residual mortality of clothianidin on mud substrates is however unclear; this requires further investigations into the characteristics of the different substrates that could affect the efficacy of different insecticides and formulations.

## Conclusion

IRS with a mixture of clothianidin and deltamethrin induced very high and prolonged mortality rates against pyrethroid resistant *An gambiae* sl owing to the clothianidin component and early exiting of mosquitoes from experimental huts due to the pyrethroid component. IRS campaigns with the insecticide mixture could significantly control malaria transmission in areas with high pyrethroid resistance.

## Supporting information

S1 Table Experimental hut data from month 1 to month 6. (XLSX)

S2 Table Experimental hut data from month 7 to month 12. (XLSX)
